# Supplementary material for: Saccharomyces boulardii (CNCM I-745) improves intestinal damage in sepsis by remodeling intestinal flora structure
Source: Front Cell Infect Microbiol. 2025 Jul 31;15:1602792. doi: 10.3389/fcimb.2025.1602792 (PMC12350329; doi:10.3389/fcimb.2025.1602792)
Supplement: Supplementary file 1 [file DataSheet1.zip › summary/summary/5_Beta_diversity/4_NMDS_result/Beta_Diversity.jaccard.NMDS.with_label.pdf]

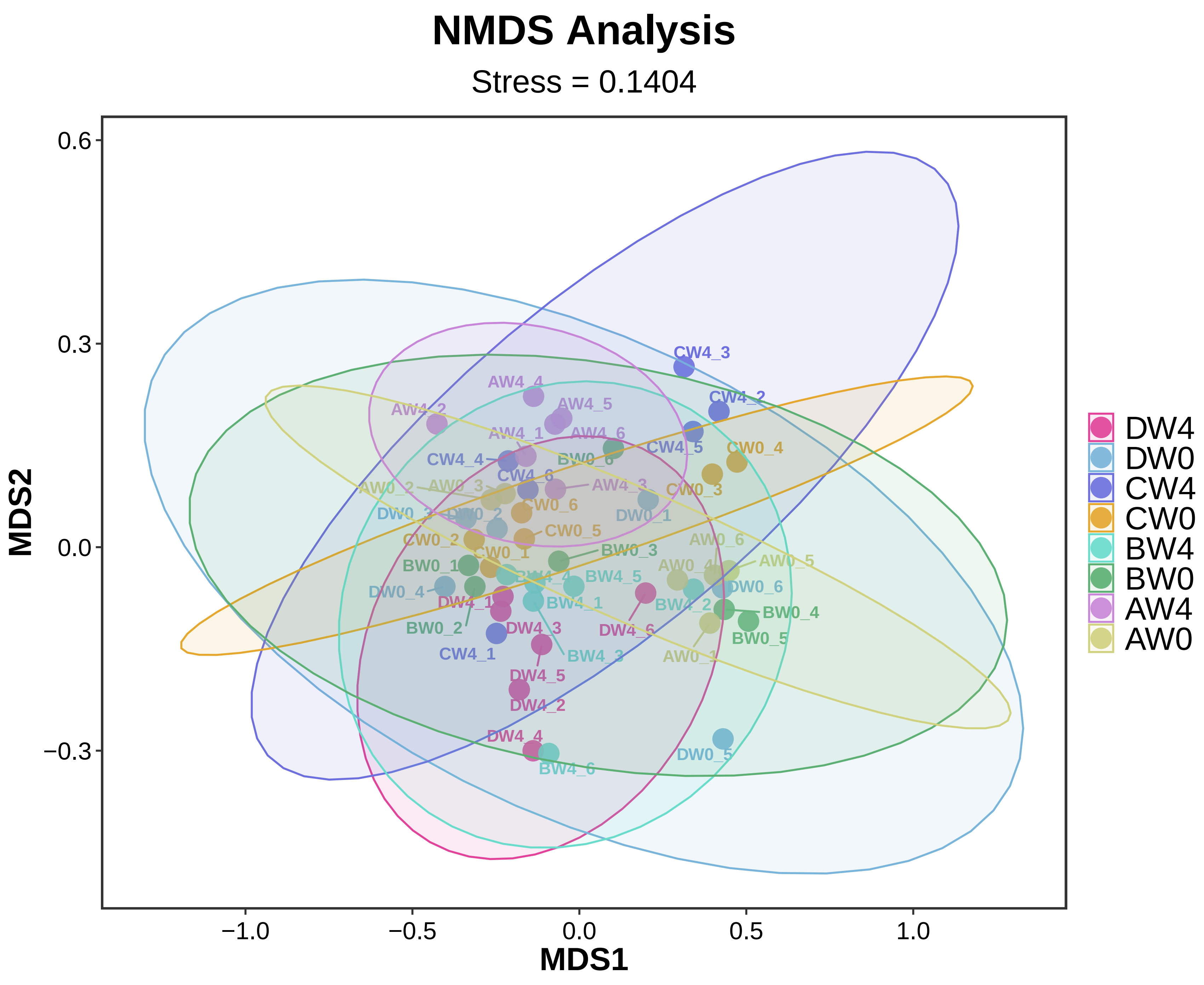

NMDS Analysis
Stress = 0.1404
MDS1
MDS2
DW4
DW0
CW4
CW0
BW4
BW0
AW4
AW0
